# Modelling G×E with historical weather information improves genomic prediction in new environments

**DOI:** 10.1093/bioinformatics/btz197

**Published:** 2019-04-12

**Authors:** Jussi Gillberg, Pekka Marttinen, Hiroshi Mamitsuka, Samuel Kaski

**Affiliations:** 1 Helsinki Institute for Information Technology HIIT, Department of Computer Science, Aalto University, Aalto, Finland; 2 Institute for Chemical Research, Kyoto University, Gokasho, Uji, Japan

## Abstract

**Motivation:**

Interaction between the genotype and the environment (G×E) has a strong impact on the yield of major crop plants. Although influential, taking G×E explicitly into account in plant breeding has remained difficult. Recently G×E has been predicted from environmental and genomic covariates, but existing works have not shown that generalization to new environments and years without access to in-season data is possible and practical applicability remains unclear. Using data from a Barley breeding programme in Finland, we construct an *in silico* experiment to study the viability of G×E prediction under practical constraints.

**Results:**

We show that the response to the environment of a new generation of untested Barley cultivars can be predicted in new locations and years using genomic data, machine learning and historical weather observations for the new locations. Our results highlight the need for models of G×E: non-linear effects clearly dominate linear ones, and the interaction between the soil type and daily rain is identified as the main driver for G×E for Barley in Finland. Our study implies that genomic selection can be used to capture the yield potential in G×E effects for future growth seasons, providing a possible means to achieve yield improvements, needed for feeding the growing population.

**Availability and implementation:**

The data accompanied by the method code (http://research.cs.aalto.fi/pml/software/gxe/bioinformatics_codes.zip) is available in the form of kernels to allow reproducing the results.

**Supplementary information:**

Supplementary data are available at *Bioinformatics* online.

## 1 Introduction

Global yield improvements are needed to feed the growing population (Tester and Langridge, 2010). One possibility is to breed varieties for higher environmental adaptability, known as *targeted breeding* (Braun *et al.*, 1996). By improving the genetic fit of varieties in their growth environments, yield potential in the interaction between the genotype and environment could be realized. Although the importance of G×E for agronomic performance is widely accepted, utilization calls for methods that predict yields in new environments, because actual experimental data, consisting of yields of plant variety candidates from yield trials, will in practice be available only from a very limited number of environments. Importantly, prediction of a plant’s response to a new environment cannot be based on weather data from the growth season, as those will never be available at the time of prediction.

Predicting the yield of a new genotype in an untested environment is an instance of ‘cold start’ problems, where predictions are needed for completely novel instances. Previously the machine learning community has developed methods for such problems, e.g. to design novel drugs for previously unseen cancers (Costello *et al.*, 2014) and to create recommendations in on-line shopping for new customers and/or products (Schein *et al.*, 2002). These methods are based on using external covariate data that describe properties of the novel instances.

**Fig. 1.**
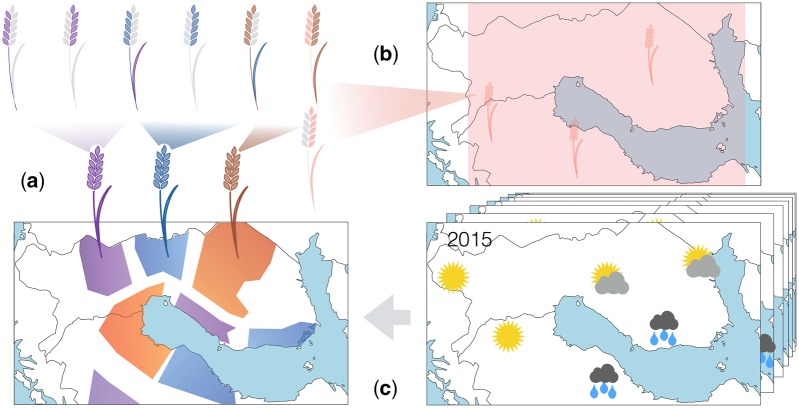
Outline of our approach. (**a**) Targeted breeding aims at producing varieties that are optimal for a specific environment, i.e. combining traits that are optimal for a particular environment in the same genotype. In the figure, traits that are optimal for each environment are illustrated as plants partially coloured according to map segments. After breeding, all optimal traits are contained in the same new genotype. When compared with traditional breeding (**b**), targeted breeding aims at higher environmental adaptation, corresponding to smaller target environments. Weather (microclimate) is a crucial driver for agronomic performance, but as it is unknown for future growth seasons, we use historical weather records (**c**) to predict the environmental stresses. The growth locations differ with respect to their estimated probabilities of growth conditions and our method can be used to manage location-specific risk: yield predictions take into account both yield potential and the susceptibility to the stresses that are most likely to occur in the environmental condition distribution at each location

We develop a new method, an extension of Kernelized Bayesian Matrix Factorization (KBMF; Gönen and Kaski, 2014), to account for the uncertainty in the covariates, which allows the use of historical records to predict weather conditions for future growth seasons, and eventually makes future G×E prediction for yield possible. Therefore, our new method, unlike the existing alternatives (Heslot *et al.*, 2014; Jarquín *et al.*, 2014; Lopez-Cruz *et al.*, 2015; Malosetti *et al.*, 2016), does not rely on accurate weather information from the growth season from the new location (Fig. 1). Although the most relevant use case for our method is making predictions for the next growing season, it could also be used to predict phenotype values in any year. For the years further in the future, however, using climate simulations instead of the historical sample of the microclimate would likely be needed.

In genomic selection (GS) (Meuwissen *et al.*, 2001), field trials are replaced with genomic predictions to speed up plant breeding. We formulate an *in silico* experimental setup for GS in targeted breeding that, unlike existing works (Albrecht *et al.*, 2014; Burgueño *et al.*, 2012; Heslot *et al.*, 2014; Jarquín *et al.*, 2017; Malosetti *et al.*, 2016; Saint Pierre *et al.*, 2016), strictly satisfies all realistic constraints: test locations, years and genotypes are all genuinely new (not part of the training set) and yields are predicted for the offspring of the training set. In this setup, we demonstrate the feasibility of targeted breeding by investigating the accuracy of G×E prediction using environmental data including historical weather information but without in-season data (Model $M_{G+E+\text{GE}}^{\text{hist}}$). We compare this with multiple competing settings, including the non-realistic ideal situation having in-season data $\left(M_{G+E+\mathrm{GE}}\right)$, a model without the G×E interaction $\left(M_{G+E}\right)$, a previous implementation with G×E interactions using in-season data (GE-BLUP) by Malosetti *et al.* (2016), and the industry standard that does not include G×E (best linear unbiased prediction using genomic data by de los Campos *et al.*, 2013, GBLUP). Data from a barley breeding programme in Finland from Boreal Plant Breeding Ltd, including historical weather information for the target environments, are divided into training, validation and test sets, and the prediction accuracy is measured as the average correlation between predicted and observed yields in the test sets (Malosetti *et al.*, 2016). A sensitivity analysis is done to further explore the impact of model assumptions.

## 2 Materials and methods

### 2.1 Data

All data used in the experiment come from a barley breeding programme in Finland, which is a part of a larger population of target environments for barley as varieties used in Finland are also used in other Nordic countries. The phenotype consists of (z-transformed) yield measurements (kg/ha) for 2244 lines observed in trials at 11 locations across the 4 southernmost growth zones in Finland from 2008 to 2015. The total number of observed *location* × *year* combinations is 35. In some locations, trials have been performed on several years and several fields with varying soil properties, and a total of 12 277 yield observations have been recorded. The number of observations per genetic line ranges from 1 to 118 (median 4). The lines were genotyped with the Illumina 9k iSelect SNP Chip, SNPs with minor allele frequency < 0.05 or with > 5% values missing were omitted. Also all genotypes with > 5% of SNPs missing were omitted. The final proportion of missing genotype data is 0.002. The experiment-specific average yields range from 3700 to 8600 kg/ha. Test fold -specific yields are summarised in Supplementary Table S3. Soil types range from organic to clay soil; climatic variation is summarized in Supplementary Figure S2.

The soil characteristics for each field block are measured in terms of the proportions of sand, silt and clay (*soil classification triangle*Shepard, 1954) and the proportion of organic content. Meteorological information consists of daily averages of temperature and rainfall, and the distances to the closest meteorological station range from 1 to 40 km (average 13.5 km). The baseline approach (GE-BLUP Malosetti *et al.*, 2016) requires summarizing the weather information per crop stage: vegetative (from sowing to visible awns), heading time (from visible awns to the end of anthesis) and grain filling (from the end of anthesis to maturity). The times of the crop stages are estimated using temperature sum accumulation; the details are given in Section 2.7. In the weather observations, the proportion of missing values in daily average temperature and rainfall measurements is < 0.0015 (max 3 missing values/environment) and < 0.0032 (max 2 missing values/environment), respectively.

### 2.2 Experimental setup

To study prediction accuracy, we use a setup that strictly imposes the realistic constraints related to modelling G×E in targeted breeding for new locations. Predictions are required for new locations (not part of the experimental grid) and for years for which no phenotype data are available (to mimic future growth seasons). Additionally, predictions are needed for the offspring of the lines in the training set, which have no phenotype data observations.

**Fig. 2.**
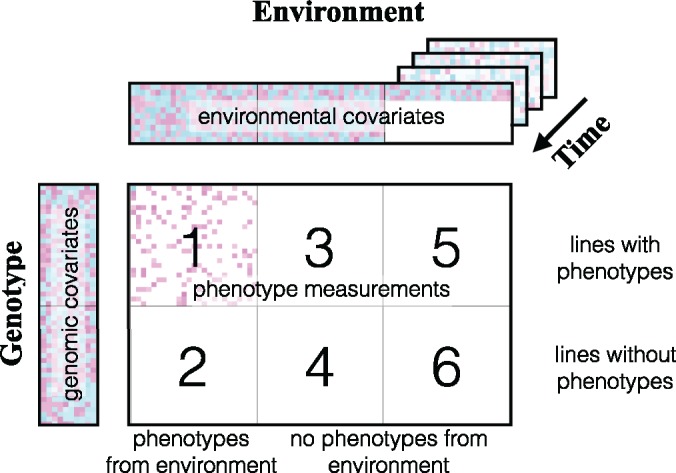
Summary of different prediction setups with respect to the availability of phenotype data and the genomic and environmental covariates as presented by Jarquín *et al.* (2017) and Malosetti *et al.* (2016). White colour indicates missing value. In Setups 1, 3 and 5, ‘lines with phenotypes’, the lines to be predicted have phenotype observations (from some environments). In Setups 1 and 2, ‘phenotypes from environment’, phenotypes have been measured from the prediction target environments (for some lines). In Setups 1–4 studied by Jarquín *et al.* (2017) and Malosetti *et al.* (2016), environmental covariates are available for all environments, whereas in the new Setups 5 and 6, environmental covariates from the trials of interest are missing and they are replaced by using several years of historical data

Different prediction tasks, distinguished by the availability of different data types, are summarized in Figure 2. Setups 1–4 have been studied by Jarquín *et al.* (2017) and Malosetti *et al.* (2016): in Setup 1, phenotype measurements are available for the genotypes and environments to be predicted, and both genotypes and environmental covariates are fully observed. In Setups 2 and 3 phenotype measurements are still available, but only for the genotypes or the environments to be predicted, but not for both, and covariates are fully observed. In Setup 4, no phenotype data are available for the environments/genotypes to be predicted, but both genetic and environmental covariates are still fully observed.

We introduce two additional setups. In Setups 5 and 6 environmental covariates from the environments of interest are only partially available: location and soil characteristics are known but in-season weather measurements are not available for the year of interest. However, historical observations for the same locations are available and can be used to estimate the performance of each genotype. Setups 5 and 6 differ in whether phenotype measurements are available from some other environment for the genotypes (5) or not at all (6). The results in this article are for the most challenging Setup 6, where no phenotype data are available for any of the lines of interest. We emphasize that a further difference to earlier work (Jarquín *et al.*, 2017; Malosetti *et al.*, 2016) is that we strictly require the test environments to simultaneously be both from a location and from a year not included among the training environments, and that the genotypes in the test and validation sets are required to be from the progeny of the training set. A summary of the differences between our setup and the earlier works is given in Supplementary Table S2.

**Fig. 3.**
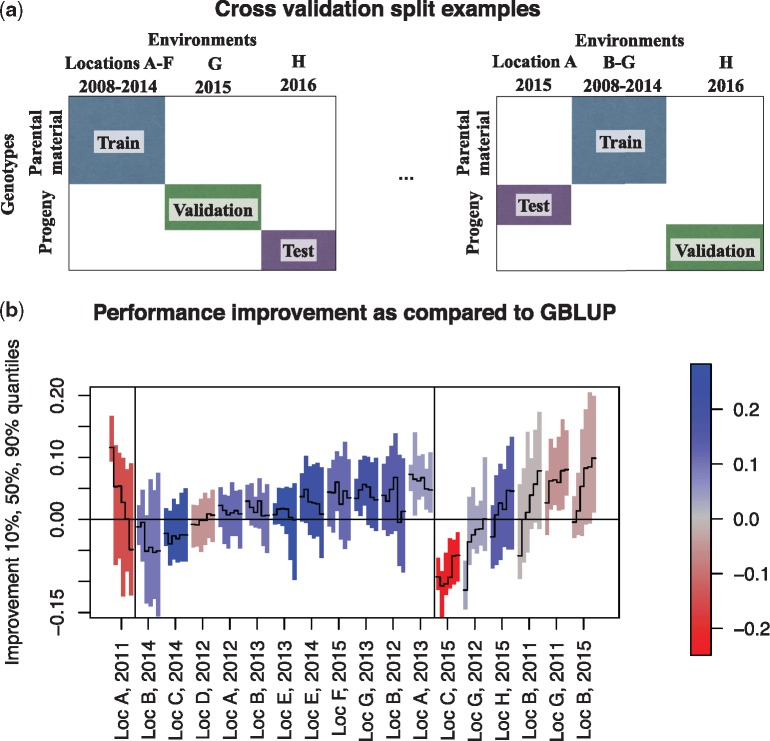
(**a**) Outline of the *in silico* setup for comparing methods. The dataset is split into training, validation and test sets so that the years, locations and genotypes in the validation and test sets are not included in the training set. (**b**) Sensitivity analysis: the difference in prediction accuracies (y-axis) between G×E prediction with historical data ($M_{G+E+\text{GE}}^{\text{hist}}$) and the industry standard (GBLUP) are shown in 18 different test environments (x-axis); values above the horizontal line mean that $M_{G+E+\text{GE}}^{\text{hist}}$ is more accurate. Six vertical bars are shown for each environment, representing variability in results (median and 90% CIs) over hyperparameter ranges, sets of genotypes that are predicted, and training-validation splits. Starting from the left, the bars correspond to models with 0, 1, 2, 3, 4 or 5 G×E interaction terms (0 corresponds to the $M_{G+E}$ model). The colour indicates the performance of GBLUP in the environment, measured as the Pearson correlation between predictions and observations and red meaning that GBLUP performed poorly (negative correlation). Results from Loc C, 2015 were omitted from the comparison as all methods performed poorly there. Vertical lines divide the environments into three groups: *left:* one environment where including G×E terms to the model decreased performance; *middle:* 11 environments where G×E terms had a neutral effect; and *right:* 6 environments where performance increased by adding more G×E terms

We measure prediction accuracy using cross-validation, where the training, validation and test sets are selected to enforce the realistic constraints (Fig. 3a). The pseudo code for the nested 3D (genotype, location and year) cross-validation is given in Supplementary Section S4. In brief, 41 test sets were constructed corresponding to different year, location and genotype sets. For each test fold, at maximum 10 training/validation set splits were further created. Hyperparameters for each test fold were selected based on the average performance over the validation sets. The final predictions were created by retraining the model with the selected hyperparameters without omitting any validation set. To maximally exploit the data in our experiment, we also used the past years as test sets (from which all data were then similarly omitted). This is made possible by the fact that the progeny genotypes were tested on several years.

To measure the prediction accuracy, we employ the commonly used Pearson correlation between the predicted and observed yields in the test set (see Albrecht *et al.*, 2014; Burgueño *et al.*, 2012; Malosetti *et al.*, 2016; Saint Pierre *et al.*, 2016). This correlation is computed for each cross-validation fold in turn, and averaged over the test cases. Similarly to Malosetti *et al.* (2016), the test-case-specific correlations are transformed into Fisher’s z-scores before averaging and back-transformed to obtain the final results. We regress the G×E interactions on the average characteristics of the growing season: for each yield trial, we use weather observations from the typical growing season (from May 1 until the end of August) regardless of the sowing date. This indirect approach enables the use of historical weather data. When predicting with historical data, the prediction for each genotype is made for each year for which historical weather observations are available, and the median of those is used as the final predicted value.

We also carry out a sensitivity analysis that allows studying the impact of modelling assumptions, such as inclusion and the number of G×E interaction components to the model. In detail, the sensitivity analysis shows variability (median and 90% interval) in the predictive performance in a given test environment (location-year combination) when we vary (i) the hyperparameter values over their specified ranges (see Section 2.4), (ii) the genotype sets that we are predicting and (iii) the training set by removing any single training environment for validation. For details of the sensitivity analysis, see Supplementary Section S7.

### 2.3 Model

In the models $M_{G+E},\;M_{G+E+\mathrm{GE}}$ and $M_{G+E+\mathrm{GE}}^{\text{hist}}$ we assume that (i) the yield *y_ij_* of genotype *i* in environment *j* is affected by the genotype, the environmental conditions throughout the growing season, and the interactions between the two, (ii) the response to the environmental properties is non-linear and that (iii) it may involve interactions between different environmental properties. For instance, temperature/rainfall either too low or too high reduces yield, and the response to rainfall is also affected by the soil type. We further assume that (iv) the responses to the environmental conditions are highly polygenic. Assumptions *i*-*iv* are encoded using the *kernel trick* (see Shawe-Taylor and Cristianini, 2004), in which covariate data are represented as similarities, or kernels, between different data items. Kernel methods are a computationally effective way to model non-linearities and interactions and they have been applied to breeding data (Gianola *et al.*, 2014). An additional complication in the data is the low number of observed trials compared with the complexity of the problem. To handle this, we constrain our model to only learn the most prominent combinations of environmental conditions affecting yield, by assuming a low-rank approximation for the model parameters accounting for the G×E effects. Finally, we follow the Bayesian statistical framework (Gelman *et al.*, 2013), and regularize the model by placing priors on all parameters, which alleviates overfitting to the training data and improves prediction accuracy in the test data.

Our method builds on KBMF (Gönen and Kaski, 2014). In brief, KBMF is a matrix factorization method that incorporates information about the dependencies between the rows and columns of the data matrix in the form of side information. The side information data sources are kernelized for efficient computation of non-linear effects. Plant breeding literature typically considers locations as static and years as a dynamic component of G×E, but we represent each environment implicitly as a probability distribution of growing conditions. We assume that a realization of that distribution fully captures the environmental covariates and hence the location and the year are not explicit factors in our regression model. These assumptions provide a framework for predicting for new years without observed weather data: an estimate for the location-specific environmental covariate distribution can be estimated using historical data and predictions are obtained by integrating over this probability distribution.

Mathematically, the model for yield is formulated as

(1) $$y_{\mathrm{ij}}=g_{i}+e_{j}+\xi_{\mathrm{ij}}+\epsilon_{\mathrm{ij}},\;i=1,\ldots ,N_{g},j=1,\ldots ,N_{e},$$

where *g_i_* is the genetic main effect, *e_j_* is the environmental effect, *ξ_ij_* is the effect that arises from interaction between genotype *i* and environment *j*, *ϵ_ij_* is noise distributed as $N(0,\sigma_{j}^{2})$, and *N*_g_ and *N*_e_ are the numbers of genotypes and environments. The genetic main effect *g_i_* is modelled as a linear function of the genomic covariates. In detail, the model for the vector of genetic main effects $g^{*}={\left(g_{1},\ldots ,g_{N_{g}}\right)}^{T}$ is given in terms of a linear genomic kernel *K*_g_ by

(2) $$\underset{N_{g}\times 1}{g^{*}}=\underset{N_{g}\times N_{g}}{K_{g}}\cdot \underset{N_{g}\times 1}{a_{g0}}+\underset{N_{g}\times 1}{e_{g0}},$$

where $a_{g0}$ are kernel regression weights and $e_{g0}$ is the noise vector with elements distributed independently as $N(0,\sigma_{g0}^{2})$. The dimension of each matrix is shown in Equation (2) below the corresponding matrix symbol. The genomic kernel *K*_g_ is computed by first concatenating the genomic covariates $g_{i}$ as the rows of a matrix **G** and then using the standard linear kernel, $K_{g}=GG^{T}$.

The environmental main effect *e_j_* in Equation (1) is modelled as a random effect,

$$e_{j}∼N(0,\sigma_{e0}^{2}),\;j=1,\ldots ,N_{e}.$$

The G×E terms *ξ_ij_* are modelled as non-linear functions of the genomic and environmental covariates, $g_{i}$ and $e_{j}$. Each environment and genotype is first represented by *R* latent variables. The interactions *ξ_ij_* are modelled as the inner product of the latent variable vectors corresponding to genotype *i* and environment *j*, i.e.

(3) $$\xi_{\mathrm{ij}}=\sum_{r=1}^{R}h_{\mathrm{ir}}^{g}\cdot h_{\mathrm{jr}}^{e},i=1,\ldots ,N_{g},j=1,\ldots ,N_{e}.$$

Here, $h_{\mathrm{ik}}^{g}$ is the *k*th latent variable for the *i*th genotype, and $h_{\mathrm{jk}}^{e}$ is the *k*th latent variable for the *j*th environment. Using matrix notation, Equation (3) can be written as

(4) $$\underset{N_{g}\times N_{e}}{\Xi }=\underset{N_{g}\times R}{H_{g}}\cdot \underset{R\times N_{e}}{H_{e}^{T},}$$

where $\Xi =\left[\xi_{\mathrm{ij}}\right]$ is the matrix of interaction terms, and $H_{g}=[h_{\mathrm{ij}}^{g}]$ and $H_{e}=[h_{\mathrm{ij}}^{e}]$ are matrices having as their rows the *R*-dimensional latent variable representations for each genotype and environment, respectively.

The latent variables *H*_g_ and *H*_e_ are obtained from genotype and environment kernels *K*_g_ and *K*_e_:

$$\underset{N_{g}\times R}{H_{g}}=\underset{N_{g}\times N_{g}}{K_{g}}\cdot \underset{N_{g}\times R}{A_{g}}+\underset{N_{g}\times R}{E_{H_{g}}}\;\text{and}$$

$$\underset{N_{e}\times R}{H_{e}}=\underset{N_{e}\times N_{e}}{K_{e}}\cdot \underset{N_{e}\times R}{A_{e}}+\underset{N_{e}\times R}{E_{H_{e}}},$$

where *A*_g_ and *A*_e_ are kernel regression weights, and $E_{H_{g}}$ and $E_{H_{e}}$ are matrices containing error terms distributed independently as $N(0,\sigma_{g}^{2})$ or $N(0,\sigma_{e}^{2})$, respectively. The environmental kernel *K_e_* is obtained by combining multiple kernels $K_{e}^{1},\ldots ,K_{e}^{E}$, computed from environmental data $e_{j},j=1,\ldots ,N_{e}$, each kernel representing a different aspect of the environment (weather, soil etc). The process for combining environmental kernels is described in Section 2.6.

Summarizing the model and introducing conjugate priors yields the distributional assumptions

$$y_{\mathrm{ij}}|H_{g},H_{e},g_{i},e_{j},\sigma_{j}^{2}∼N(g_{i}+e_{j}+{\left(h_{i}^{g}\right)}^{T}h_{j}^{e},\sigma_{j}^{2}),\;\forall (i,j)$$

$$\sigma_{j}^{-2}∼G(\alpha_{j},\beta_{j}),\;\forall (j)$$

$$a_{i}^{g0}|\lambda_{g0}∼N(0,\lambda_{g0}^{-1}),\;\forall (i)$$

$$g_{i}|a_{g0},K_{g},\sigma_{g0}^{2}∼N(a_{g0}^{T}k_{i}^{g},\sigma_{g0}^{2}),\;\forall (i)$$

$$a_{\mathrm{ij}}^{g}|\lambda_{g}∼N(0,\lambda_{g}^{-1}),\;\forall (i,j)$$

$$h_{\mathrm{ij}}^{g}|A_{g,}K_{g},\sigma_{g}^{2}∼N({\left(k_{i}^{g}\right)}^{T}a_{j}^{g},\sigma_{g}^{2}),\;\forall (i,j)$$

$$e_{j}|\sigma_{e0}^{2}∼N(0,\sigma_{e0}^{2}),\;\forall (j)$$

$$a_{\mathrm{ij}}^{e}|\lambda_{e}∼N(0,\lambda_{e}^{-1}),\;\forall (i,j)$$

$$h_{\mathrm{ij}}^{e}|A_{e},K_{e},\sigma_{e}^{2}∼N({\left(k_{i}^{e}\right)}^{T}a_{j}^{e},\sigma_{e}^{2}),\;\forall (i,j),$$

where $k_{i}^{g},\;k_{j}^{e},\;a_{j}^{g}$, $a_{j}^{e}$, denote columns of matrices *K*_g_, *K*_e_, *A*_g_, *A*_e_, with subscripts *i* and *j* specifying the column index; $h_{i}^{g}$ and $h_{j}^{e}$ denote *i*th and *j*th rows of *H*_g_ and *H*_e_, represented as column vectors; $a_{i}^{g0}$ is the *i*th element of vector $a_{g0}$; $a_{\mathrm{ij}}^{g}$ and $a_{\mathrm{ij}}^{e}$ are the (*i*, *j*)th elements in matrices *A*_g_ and *A*_e_. N and G denote the Gaussian and Gamma distributions, respectively.

### 2.4 Specifying hyperparameter values

We use a combination of prior knowledge and cross-validation to determine the hyperparameters: first prior knowledge to inform about a grid of sensible values, then cross-validation to select a value from the grid. To express the prior knowledge, we relate the sizes of the different terms in Equation (1) to the total variation of the output variables *y_ij_* and to each other, and select hyperparameters such that the expected proportion of total variance explained (PTVE) scales reasonably with the total variance of the outputs; e.g. the covariates obviously cannot explain >100% of the variance. In practice, because the PTVE depends also on the data, its value corresponding to a given hyperparameter combination is estimated using Monte Carlo simulation. This way, either a single fixed value or a grid of values to be selected from by cross-validation is determined for each hyperparameter (see below for details). Previously, a similar approach has been used by Gillberg *et al.* (2016). The assumptions about the relative sizes of different effects that were used to define the numeric values of the hyperparameters are given below.

Parameters $\left(\alpha_{j},\beta_{j}\right)$ of the Gamma distribution for environment-specific residual noise variances $\sigma_{j}^{2}$ are set to (10, 1), corresponding to an expected value of ∼0.1 for $\sigma_{j}^{2}$ (recall that the variance of each output is equal to unity). The variance of environment mean effects $\sigma_{e0}^{2}$ is fixed to 0.25. To set the parameters $\lambda_{g0}$ and $\sigma_{g0}^{2}$ that determine the amount of signal and noise in the genetic main effects, we find values for them such that two conditions are satisfied. First, for pragmatic purposes, 95% of the variance of the genetic effects $g^{*}$ is assumed to be signal, i.e.

$$\frac{\text{Var}(K_{g}\cdot a_{g0})}{\text{Var}(K_{g}\cdot a_{g0})+\sigma_{g0}^{2}}=0.95.$$

This large value maintains the identifiability of the different terms in Equation (1), by assuming that noise comes mainly from the last term *ϵ_ij_*. However, the value cannot be made exactly equal to unity, to have some flexibility in the variational inference algorithm. The second condition is that the variance of the genetic main effects, Var_genetic_ = Var$(K_{g}\cdot a_{g0})+\sigma_{g0}^{2}$, is either 0.2, 0.4 or 0.6, to be selected by cross-validation.

The parameters $\lambda_{g},\sigma_{g}^{2},\lambda_{e},$ and $\sigma_{e}^{2},$ controlling the proportion of signal and noise in the latent components *H*_g_ and *H*_e_ are selected similarly: *H*_g_ and *H*_e_ model the G×E interactions and the values of $\lambda_{g},\sigma_{g}^{2},\lambda_{e}$ and $\sigma_{e}^{2},$ were determined by inspecting the proportion of signal of the total variance of the latent factors and the relative contribution of the interaction terms compared with the main genetic effects. In detail, we first assume that

$$\frac{\text{Tr}(\text{Var}(K_{g}\cdot A_{g}))}{\text{Tr}(\text{Var}(K_{g}\cdot A_{g}))+R\sigma_{g}^{2}}=0.95,\;\text{and}$$

$$\frac{\text{Tr}(\text{Var}(K_{e}\cdot A_{e}))}{\text{Tr}(\text{Var}(K_{e}\cdot A_{e}))+R\sigma_{e}^{2}}=0.95,$$

where Tr() denotes the trace of a matrix. Second, we assume that the total variance of the interactions is either the same or half of the total variance from the genetic main effects, i.e.

$$\text{Tr}(\text{Var}(H_{g}\cdot H_{e}^{T}))=\Phi \times R\times [\text{Var}(K_{g}\cdot a_{g0})+\sigma_{g0}^{2}],$$

where Φ is either 0.5 or 1, to be selected by cross-validation.

### 2.5 Inference

For inference we use variational approximation (Beal, 2003), which is a computationally feasible way to approximate posteriors of parameters in complex models. The variational updates required here can be derived similarly to Gönen and Kaski (2014), except that we have extended their model and algorithm by including the genotype and environment main effects, i.e. the terms *g_i_* and *e_j_* in Equation (1). Further details are given in Supplementary Section S2.

### 2.6 Data pre-processing and kernels

A summary of different data source specific kernels, pre-processing and transformations, is given in Supplementary Table S1. The bandwidth parameter of the Gaussian kernels is set to the default value equal to the number of covariates used to compute the kernel. All kernels *K* are normalized to make them unit diagonal:

(5) $$\tilde{K}=(d^{-1/2}\times d^{-1/2})\cdot K,$$

where **d** is a vector of the diagonal values of kernel *K*, × denotes the outer product, and the $d^{-1/2}$ denotes a vector with all elements of **d** raised to the power of –½. The interaction kernel between the soil type and rainfall is computed from other kernels as

(6) $$K_{\text{soil}\;x\;\text{rain}}={\tilde{K}}_{\text{soil},\;\text{Gaussian}}⊙{\tilde{K}}_{\text{rain},\;\text{Gaussian}},$$

where ⊙ denotes the Hadamard (elementwise) product. Finally, all kernels are normalized with respect to their summed total variance by multiplication with a constant *c*

(7) $$\tilde{\tilde{K}}=c\cdot \tilde{K}$$

where $c={\left[\sum_{i=1}^{N}\text{Var}({\tilde{k}}_{i})\right]}^{-1/2}$ and ${\tilde{k}}_{i}$ is the *i*th column of $\tilde{K}$. This normalization enforces the expectation that, when multiple kernels are combined as described below, each kernel explains a priori the same amount of variance.

The environmental kernel *K*_e_ is formulated as a weighted sum of data source and transformation specific normalized kernels ($\tilde{\tilde{K}}$), see Supplementary Table S1. The weights reflect the importance of data sources, and they are learned by fitting Bayesian Efficient Multiple Kernel Learning (BEMKL; Gönen, 2012), a multiple kernel regression method, to the training data using experiment-specific yield means as the target variable. For BEMKL, shape (*α*) and scale (*β*) parameters of the Gamma priors are set to 1, except for the *λ* parameter, whose scale is fixed to 10 to provide stronger regularization. Regression bias term *b* is set to 0. For details of BEMKL, see Gönen (2012). Before combining the kernels, the weights are normalized such that their sum of squares is equal to 1 and the largest weight (in absolute value) is positive. Results of the sensitivity analysis of the kernel weights are presented in Supplementary Figure S1. Finally, the composite kernel *K*_e_ is normalized according to Equation (7).

### 2.7 Comparison methods

The mixed model computations for the comparison methods GBLUP and GE-BLUP are performed using the R library rrBLUP (Endelman, 2011). For both methods, fixed effects were used to account for field block-specific effects, corresponding to the terms *e_j_* in $M_{G+E+\text{GE}}^{\text{hist}},\;M_{G+E+\text{GE}}$ and $M_{G+E}$. For GBLUP, the genomic kernel (see Section 2.3) was used as the covariance matrix Σ. For GE-BLUP, the environmental kinship model (model GE-KE in Malosetti *et al.*, 2016), is used and the full covariance matrix Σ is generated through the Kronecker product $\Sigma =\Sigma_{G}⊗\Sigma_{E}$, where Σ_G_ and Σ_E_ are the genetic and environmental covariance matrices, respectively. The environmental covariance matrix Σ_E_ is generated from the available environmental data to describe soil properties and the growth conditions during the vegetative, heading time and grain filling developmental stages. All soil data and growth zone information are used as such whereas the daily average temperature and rainfall measurements are summarized as the mean and the standard deviation of the daily observations per crop stage. The growth periods are estimated using the sowing date and temperature sum accumulation-based estimates of heading and ripening times (440.2 and 905.9°C, respectively), which were estimated from external breeding data. The vegetative stage is assumed to last 3 weeks starting from sowing, the time of heading is assumed to start 2 weeks before and last 1 week after the estimated heading time and grain filling was assumed to start after heading and to last 1 week longer than the estimated time of ripening. Wide estimates for the growth periods were used to account for varying growth speeds. The resulting set of environmental covariates is z-normalized and a linear kernel is used, further normalized according to Equation (5).

## 3 Results

Table 1 shows a comparison of the different methods in the challenging setup where yield experiments are not available for either the genotypes or the environment (i.e. location-year combination). We see that modelling G×E with historical weather data, $M_{G+E+\text{GE}}^{\text{historical}}$, improves prediction accuracy as compared with the industry standard, GBLUP (*P* = 0.023, a two-sided paired Wilcoxon signed rank test, df = 17). The improvement is comparable to using in-season data ($M_{G+E+\text{GE}}$, *P* = 0.011). The Bayesian methods in general show a higher accuracy whereas GE-BLUP performs poorly with the data available. Overall, the absolute prediction accuracy of all methods is relatively low in this challenging setup, with $M_{G+E+\text{GE}}^{\text{hist}}$ having the highest correlation of 0.105. Nevertheless, the improvement is considerable over the industry-standard with correlation 0.077, which corresponds to the proposed new method explaining 85% more of the variation of the phenotype on average.

**Table 1.** Comparison of prediction performance in different setups

	New location, year and genotype			CV1: new genotype, tested environment			CV2: tested genotype, new environment		
Model	Mean	SE	*P*	Mean	SE	*P*	Mean	SE	*P*
$M_{G+E+\text{GE}}$	0.104	0.04	0.011	0.173	0.040	0.16	0.251	0.034	0.22
$M_{G+E+\text{GE}}^{\text{hist}}$	0.105	0.03	0.023	0.158	0.042	0.64	0.244	0.034	0.81
GE-BLUB	0.004	0.03	0.109	0.123	0.04	0.458	0.191	0.036	0.10
GBLUB	0.077	0.03	N/A	0.156	0.038	N/A	0.240	0.034	N/A
$M_{G+E}$	0.097	0.04	0.109	0.151	0.040	0.75	0.237	0.034	0.71

*Note*: The new prediction method outperforms the others in the most challenging setup and performs equally in the less demanding setups. *Mean*, correlation between predicted and observed yields, averaged across test sets; SE, standard error of the mean; *P, P*-value compared with the industry standard (GBLUP).

In the less challenging setups CV1 and CV2, where measurements are available for either the genotype or the environment, the Bayesian methods that incorporate environmental information achieve a minor improvement, which is not statistically significant in prediction accuracy as compared with the comparison method GBLUP (see Table 1). The improvement is not as dramatic as has been reported earlier (Heslot *et al.*, 2014; Jarquín *et al.*, 2014; Malosetti *et al.*, 2016). The explanation for this may be our indirect way of using the environmental data, by considering weather data over the whole growing period regardless of the exact sowing date, which may not be as effective for this problem as the models formulated by others. This hypothesis could be studied by the means of a direct comparison with the methods from e.g. Heslot *et al.* (2014); Jarquín *et al.* (2014) on such a dataset, which contains more accurate information about the growing stages of the varieties, as required by the earlier methods.

Comparison of the results from the proposed new setup, CV1 and CV2 well quantifies the relative challenges related to the different setups. Predicting the performance of a new genotype in a tested environment is considerably more challenging (average correlation between predictions and test data ∼0.16) than predicting the performance of a line for which phenotype observations are already available (average correlation ∼0.24), and simultaneously making predictions for new environments and new genotypes is still substantially more difficult (average correlation ∼0.08), as was also observed by Jarquín *et al.* (2017) and Malosetti *et al.* (2016). Clearly the used *in silico* experimental setup dominates the general level of predictive performance and should be chosen carefully when evaluating computational methods for plant breeding. Finally, the needs for processing environmental data are different in the three setups. In CV1 and CV2 methods have access to phenotype data from all the environments and G×E effects can be modelled implicitly without explicitly taking into account environmental data. We hypothesize that as a result, to gain significant performance improvements, accurate data is needed. In the new setup, the environment is completely new and transfer of information about variety performance under the conditions of the new environment calls for explicit modelling of G×E.

The sensitivity analysis demonstrates considerable variability between test environments (Fig. 3b). Indeed, including G×E interaction terms into the model decreased accuracy in 1/18 environments, had little effect in 11/18 environments, but improved the accuracy substantially in 6/18 environments. In the last group, increasing model complexity by adding more G×E components consistently improved performance, which highlights the potential to increase accuracy through complex modelling of G×E. Importance of different data sources to the predictions can be further analysed by investigating the kernel weights that the method has learned to summarise the contributions of the data sources (Supplementary Fig. S1). We see that the two most influential kernels were the ones representing (i) the non-linear interaction between soil type and daily rainfall, and (ii) the non-linear effect of rain, matching well the biological understanding of the problem.

## 4 Discussion

Our experiments confirmed that prediction in new environments is a challenging task, as reported earlier (Jarquín *et al.*, 2017; Malosetti *et al.*, 2016), our new method reaching the highest correlation of 0.105 between predictions and observations. Nevertheless, the usefulness of including multiple G×E interaction terms and non-linear interactions between environmental covariates became evident from our results. We expect that gains from modelling G×E will increase in the future as more data, representing further locations and years, will allow more accurately distinguishing the interactions from the main effects. Other ways to improve the predictions include using more detailed genomic modelling, e.g. using Gaussian and other kernels for summarizing the SNP data.

Besides targeted breeding, there are several other needs for G×E prediction models. They could mitigate the problems of conventional breeding: accounting for historical weather in the actual target population of environments can help prevent overfitting to the conditions in the few field trials performed, as discussed in detail in Supplementary Section S3. The assumption of the match between field trials and actual growing locations is equally crucial for the official variety trials for value of cultivation and use, required in most countries to evaluate new varieties. G×E models are also needed in assessing the effects of climate change and to select for varieties that react favourably to the altering conditions (Tester and Langridge, 2010). For this purpose, the historical weather observations in $M_{G+E+\text{GE}}^{\text{hist}}$ can be replaced with climate simulations to assess the performance of varieties under various climate scenarios.

To summarize, we showed that G×E prediction in the setup required by targeted breeding, where the environments are strictly new and predictions are based on historical weather data available at the time of prediction, significantly outperforms the current industry standard in prediction accuracy. Such improvements are essential to accelerate the implementation of targeted breeding. Future work includes comparing methods for G×E prediction on datasets comprising all measurements required by the different methods, facilitating a meaningful comparison. Adopting techniques that are currently being studied in the wider context of G×E research, such as marker × environment interactions (Cuevas *et al.*, 2016; Lopez-Cruz *et al.*, 2015) and modelling multiple traits simultaneously (Dias *et al.*, 2017; Montesinos-López *et al.*, 2016), could further improve the usefulness of our approach.

## Supplementary Material

btz197_Supplementary_Data
