# Supplementary material for: Modelling G × E with historical weather information improves genomic prediction in new environments
Source: Bioinformatics. 2019 Apr 12;35(20):4045–52. doi: 10.1093/bioinformatics/btz197 (PMC6792123; doi:10.1093/bioinformatics/btz197)
Supplement: btz197_Supplementary_Data [file btz197_supplementary_data.pdf]

Supplementary Information (SI)

1 Details of the environmental data processing and estimated kernel weights

Supplementary Figure S 1 presents the weights estimated for each environmental data source. Table S 1 presents the preprocessings that were applied to the different environmental covariates and the kernel functions used.

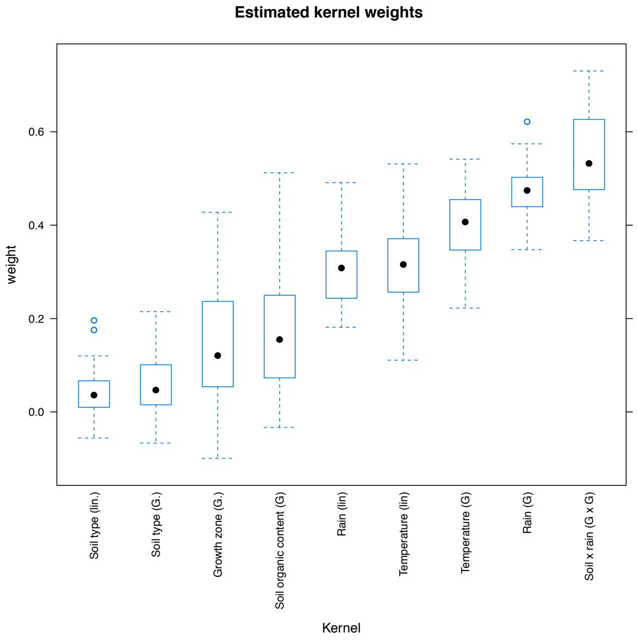

Fig. S 1. Sensitivity analysis of the estimated normalized kernel weights.

2 Details of the variational inference algorithm

For short-hand, the hyper-parameters in the model are denoted jointly by

$$\zeta = \{\alpha_j, \beta_j, \sigma^2_{g0}, \sigma^2_{e0}, \sigma^2_g, \sigma^2_e, \lambda_{g0}, \lambda_g, \lambda_e\},$$

and the parameters by

$$\Theta = \{\mathbf{a}_{g0}, A_g, A_e, H_g, H_e, \mathbf{g}^*, \mathbf{e}^*, \sigma^2_*\},$$

where $\sigma^2_* = (\sigma^2_1, \ldots, \sigma^2_{N_e})$. In the following the dependence on ζ is omitted for clarity. We assume the factorized variational approximation

$$p(\Theta|K_g, K_e, Y) \approx q(\Theta) = q(\mathbf{a}_{g0})q(A_g)q(A_e)q(H_g)q(H_e)q(\mathbf{g}^*)q(\mathbf{e}^*)q(\sigma^2_*)$$

and define each factor in the ensemble just like its full conditional:

$$q(\mathbf{a}_{g0}) = \mathcal{N}(\mathbf{a}_{g0}; \mu(\mathbf{a}_{g0}), \Sigma(\mathbf{a}_{g0}))$$

$$q(A_g) = \prod_{r=1}^{R} \mathcal{N}(\mathbf{a}^g_r; \mu(\mathbf{a}^g_r), \Sigma(\mathbf{a}^g_r))$$

$$q(A_e) = \prod_{r=1}^{R} \mathcal{N}(\mathbf{a}^e_r; \mu(\mathbf{a}^e_r), \Sigma(\mathbf{a}^e_r))$$

$$q(H_g) = \prod_{i=1}^{N_g} \mathcal{N}(\mathbf{h}^g_i; \mu(\mathbf{h}^g_i), \Sigma(\mathbf{h}^g_i))$$

$$q(H_e) = \prod_{j=1}^{N_e} \mathcal{N}(\mathbf{h}^e_j; \mu(\mathbf{h}^e_j), \Sigma(\mathbf{h}^e_j))$$

$$q(\mathbf{g}^*) = \prod_{i=1}^{N_g} \mathcal{N}(g_i; \mu(g_i), \Sigma(g_i))$$

$$q(\mathbf{e}^*) = \prod_{j=1}^{N_e} \mathcal{N}(e_j; \mu(e_j), \Sigma(e_j))$$

$$q(\sigma^2_*) = \prod_{j=1}^{N_e} \mathcal{G}(\sigma_j^{-2}; \alpha(\sigma_j^{-2}), \beta(\sigma_j^{-2})).$$

The parameters in the factor distributions can be derived as by Gönen and Kaski (2014), and they are therefore omitted from here.

Initialisation of the variational algorithm. The parameter $\mathbf{g}^*$ was initialised to the main genetic effects learnt by `GBLUP`, and $\mathbf{e}^*$ was initialised to the average yields in the different environments. Parameters H_g and H_e were initialised by applying the regularized Singular Value Decomposition (SVD) implemented in R library `softImpute` to the yield matrix Y after regressing out the initialised main effects $\mathbf{g}^*$ and $\mathbf{e}^*$. Parameters $\mathbf{a}_{g0}$, A_g and A_e were initialised to 0. Environment-specific residual variance parameters σ^2_* were initialised to environment-specific sample variances.

3 Gains from modelling $G \times E$ for current target population of environments

Our results indicate targeted breeding could improve yields by dividing a single target population of environments (TPE) into several parts, but the same methodology could be used even when developing only 1 variety for a larger population of target environments as in traditional breeding. Traditional breeding makes the implicit assumption that varieties' observed yields $g \in 1, \ldots, G$ in trial experiments in environments (location $\times$ year) $e \in 1, \ldots, E$, are representative of the yield in the TPE, in other words

$$p(\text{yield}_g|\text{TPE}) \approx \frac{1}{E} \sum_e p(\text{yield}_g|\text{environment}_e) \quad (1)$$

However, with geographic field use information and weather data widely available, this strong assumption can be replaced with an estimate for the yield in the TPE given the actual fields and their microclimates:

$$p(\text{yield}_g|\text{TPE}) \approx \sum_f^F P_f \times p(\text{yield}_g|f) \quad (2)$$

$$= \sum_f^F P_f \times \int_{\theta_f} p(\text{yield}_g|\theta_f) \times p(\theta_f) d\theta_f, \quad (3)$$

where $f \in 1, \ldots, F$, are fields in the TPE used for cultivation of the new variety, θ_f are parameters (e.g. weather conditions) related to a certain

Variable (unit)	transformation	preprocessing parameters	missing value imputation	kernel transformation(s)
Soil content (%, N_{covs} =3)	log transformation	z-normalization	(none)	linear and Gaussian
Soil organic content (%, N_{covs} =3)	log transformation	z-normalization	(none)	Gaussian
daily rainfall (mm, N_{covs} =123)	7-day moving average (6 previous days)	z-normalization with 3rd order polynomial smoothing	0-imputation	linear and Gaussian
daily average temperature (C°, N_{covs} =12 3)		z-normalization with 3rd order polynomial smoothing of daily mean/scale parameters	0-imputation	linear and Gaussian
growth zone (1-4, N_{covs} =1)		z-normalization	(none)	Gaussian
genotype markers (SNPs, N_{covs} =5696)		Minor allele frequency scaling for SNP A: $\frac{\mathrm{A} - 2 \cdot \mathrm{MAF_A}}{\sqrt{2 \cdot \mathrm{MAF_A} \cdot (1 - \mathrm{MAF_A})}}$	mean imputation	linear kernel

Table S 1. Preprocessings and kernel functions applied to covariates.

field f, $p(\theta_f)$ is the uncertainty related to these conditions, estimated from historical records, $p(\text{yield}_g|\theta_f)$ is the predictive distribution for the yield under conditions θ_f, obtained from the model, and P_f is the proportion of the total volume cultivated in field f.

4 Details of the cross validation scheme

The data consists of two generations of lines: a parental and progeny generation. Data is available from several years and locations.

Algorithm 1 presents the pseudo code for the 3D nested cross validation used to evaluate performance in new location, on new years for new genotypes.

Table S2 presents a detailed comparison of the proposed cross-validation setup to earlier works.

5 Climatic variation between the trial locations

Statistics about the weather conditions on trial locations on different months and years is presented in Figure S 2.

6 Details of the cross validation split

Table S 3 presents statistics about the cross validation splits.

7 Details of the sensitivity analysis

In the sensitivity analysis, the effect of the removal of training environments (removing all data from one further location and year from the training set) is obtained by evaluating test set performance with the models trained during cross validation after omitting different validation folds (line 22 in pseudo code): test set performance was evaluated with each of these models to measure the sensitivity of model performance to the decomposition of the training set.

The number of test folds and validation folds corresponding to each location-year combination varies (see Table S 3) due to availability of observations in the data set. The total number of validation folds for the 41 test folds is 343. Information about training and test set sizes and the number of validation folds for different cross validation splits is given in Supplementary Table S 3.

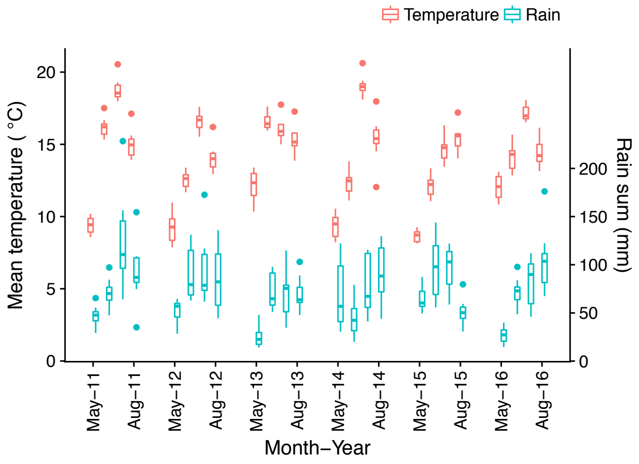

Fig. S 2. Climatic variation among the different trial locations on different months and years. The dashed lines denote the mean ± standard deviation.

Algorithm 1 3D Nested Cross Validation for New Year, New Location and New Genotype -performance estimation

L: set of all locations
Y: set of all years
$G_{parental}$: set of all lines in the parental population
$G_{progeny}$: set of all lines in the progeny population
Θ: set of model hyperparameter combinations
Initialise training set (all data), validation set (empty set) and test set (empty set)
Assign test set:
for test location in L **do**
  for test year in Y **do**
    for set of test genotypes in $G_{progeny}$ **do**
      Assign all observations from { test location, test year and set of test genotypes} as the test set.
      Omit all other data from the test year, location or lines from the training set (not only combination, each condition individually)
      Assign validation set:
      for validation location in $\{L \setminus \text{test location}\}$ **do**
        for validation year in $\{Y \setminus \text{test year}\}$ **do**
          for set of validation genotypes in $G_{progeny} \setminus$ test genotypes **do**
            Assign all observations from { validation location, validation year and set of validation genotypes} as the validation set.
            Omit all other data from the validation year, location or lines from the training set (not only combination, each condition individually)
            Omit all other data $G_{progeny}$ from the training set
            for model hyperparameters θ in Θ **do**
              Train model on training set
              Compute performance on validation set
            end for
          end for
        end for
      end for
      Select hyperparameter combination by averaging over the different validation sets.
      Assign training set without omitting any validation set, however, omit all data from $G_{progeny}$
      Train model on the training set
      Compute performance on the test set
    end for
  end for
end for
Estimate generalisation performance by averaging over the different test sets.

Publication	New environment	New genotypes
Burgueño *et al.* (2012) (CV1/CV2)	CV1/CV2: test locations and years are present in the location-year combinations in the training data	new lines in CV1: not restricted to the offspring generation. In CV2 the test lines have phenotype observations
Heslot *et al.* (2014)	Random split, balanced wrt years and locs $\rightarrow$ years and locations not new	only 544/2195 genotypes have no phenotype observations, test set not restricted to the offspring generation
Albrecht *et al.* (2014)	the year-location combination is new but the test locations and years are present in other location-year combinations in the training data	genotypes are new and from the offspring
Malosetti *et al.* (2016)	time-structured DTD: 2/6 test locations new according to strict criteria; physically structured DTD: none of the environments are strictly new (as the year is not new)	all genotypes within the same family, not from the next generation.
Saint Pierre *et al.* (2016) (leave-one-side-out)	location new but year part of the training set	test lines have phenotype observations
Jarquín *et al.* (2017)	CV00: new location-year combination but the location and year are present in other combinations.<br>CV0: new location or year	CV00: new genotypes but not from the offspring generation. CV0: phenotype observations available for the genotypes

Table S 2. Comparison of the proposed in silico setup to the existing setups.

	environment	n phenotypes final training set	n validation folds	n phenotypes nested CV mean ± sd	n phenotypes validation mean ± sd	yield mean ± sd	n phenotypes test set
1	Loc B, 2011	5676	10	4197 ± 557	113 ± 46	6685 ± 585	59
2	Loc A, 2011	7049	10	4778 ± 998	113 ± 49	5509 ± 741	59
3	Loc G, 2011	6615	10	4519 ± 953	121 ± 57	6054 ± 620	58
4	Loc B, 2013	5205	9	4028 ± 492	95 ± 58	5061 ± 565	182
5	Loc A, 2012	7294	10	4977 ± 1084	94 ± 36	5799 ± 829	106
6	Loc G, 2012	6709	10	4529 ± 1042	94 ± 36	5186 ± 745	106
7	Loc D, 2012	8406	10	5994 ± 1072	96 ± 38	3617 ± 667	105
8	Loc E, 2013	6990	10	4972 ± 798	103 ± 71	7178 ± 589	91
9	Loc B, 2012	5613	10	4168 ± 557	97 ± 39	4735 ± 768	106
10	Loc G, 2013	5902	10	4086 ± 823	105 ± 67	5223 ± 1025	91
11	Loc B, 2012	5613	8	4126 ± 559	80 ± 21	5414 ± 768	260
12	Loc A, 2012	7294	10	4854 ± 1038	90 ± 36	5760 ± 780	243
13	Loc E, 2013	6990	10	4972 ± 746	84 ± 23	6948 ± 752	153
14	Loc G, 2013	5902	10	4102 ± 818	86 ± 23	5557 ± 811	152
15	Loc C, 2014	8391	10	5786 ± 1018	89 ± 35	3978 ± 481	79
16	Loc B, 2013	5205	9	3891 ± 517	86 ± 23	5932 ± 491	153
17	Loc A, 2013	6141	10	4293 ± 828	86 ± 23	7096 ± 766	153
18	Loc E, 2014	8231	10	5590 ± 953	89 ± 36	4858 ± 556	79
19	Loc B, 2014	5769	9	4022 ± 696	83 ± 21	4408 ± 398	79
20	Loc B, 2014	5769	10	3816 ± 631	120 ± 53	4130 ± 637	106
21	Loc C, 2014	8391	10	5491 ± 888	130 ± 61	4886 ± 784	106
22	Loc E, 2014	8231	10	5352 ± 795	131 ± 64	5378 ± 852	105
23	Loc H, 2015	7450	10	4792 ± 933	122 ± 59	4411 ± 379	64
24	Loc F, 2015	8205	10	5524 ± 935	122 ±59	7287 ± 694	64
25	Loc B, 2015	5610	10	3778 ± 682	113 ± 51	5699 ± 903	64
26	Loc B, 2013	5731	6	4758 ± 371	68 ± 19	5167 ± 1095	488
27	Loc G, 2013	6703	9	4805 ± 1029	68 ± 20	5544 ± 892	244
28	Loc E, 2013	7996	8	6036± 1017	65 ± 19	7175 ± 782	244
29	Loc C, 2014	9393	5	7190 ± 1273	55 ± 12	5232 ± 499	120
30	Loc F, 2015	9409	3	7546 ± 1493	91 ± 0	7053 ± 811	39
31	Loc E, 2014	9172	6	7034 ± 1275	56 ± 11	5600 ± 459	120
32	Loc B, 2015	6432	2	5642 ± 276	91 ± 0	7463 ± 489	39
33	Loc B, 2014	6504	6	5212± 891	119 ± 97	3571 ± 591	91
34	Loc E, 2014	9172	8	6748 ± 1393	133 ± 158	5485 ± 791	91
35	Loc C, 2014	9393	8	6838 ± 1422	156 ± 160	4494 ± 606	91
36	Loc H, 2015	8652	5	6170 ± 1526	243 ± 150	4013 ± 554	42
37	Loc B, 2015	6432	4	4881 ± 1037	182 ± 72	6537 ± 659	42
38	Loc B, 2015	6432	6	5135 ± 903	152 ± 73	7829 ± 596	64
39	Loc F, 2015	9409	8	7159 ± 1439	186 ± 138	6167 ± 924	64
40	Loc H, 2015	8652	8	6402 ± 1439	186 ± 138	7224 ± 671	63
41	Loc C, 2015	9111	6	6531± 1445	213 ± 152	5356 ± 1024	60

Table S3. Statistics about the different training, validation and test splits used in the cross validation.
